# Relationship between blood pressure reverse dipping and type 2 diabetes in hypertensive patients

**DOI:** 10.1038/srep25053

**Published:** 2016-04-25

**Authors:** Lu Sun, Bin Yan, Ya Gao, Dan Su, Liyuan Peng, Yang Jiao, Yuhuan Wang, Donggang Han, Gang Wang

**Affiliations:** 1 Department of Ultrasound, the Second Affiliated Hospital, Xi’an Jiaotong University, Xi’an, China; 2 Department of Emergency Medicine, the Second Affiliated Hospital, Xi’an Jiaotong University, Xi’an, China; 3 Department of Cardiology, the Second Affiliated Hospital, Xi’an Jiaotong University, Xi’an, China; 4 Department of Endocrinology, the Second Affiliated Hospital, Xi’an Jiaotong University, Xi’an, China

## Abstract

Recent studies suggested that nocturnal variations of blood pressure (BP) were closely related to type 2 diabetes. However, little information has been revealed about the relationship between reverse-dipper pattern of BP and type 2 diabetes. In this cross-sectional study, BP variations of 531 hypertensive patients were evaluated with ambulatory BP monitoring (ABPM). Diagnosis of diabetes in Chinese adults was made according to diabetes diagnostic criteria of 2015. Multivariate logistic regression was used to examine the relationships between type 2 diabetes and ABPM results. In the study, patients with reverse-dipper pattern (32.3%) had the highest prevalence of type 2 diabetes compared with dippers (21.4%) and nondippers (23.3%). After multivariate logistic regression, reverse-dipper BP pattern (OR 2.067, *P* = 0.024) and nondipper BP pattern (OR 1.637, *P* = 0.039) were found to be correlated with type 2 diabetes compared with dipper pattern. The results of our study also suggested that type 2 diabetes might contribute to the reverse-dipper pattern of BP (OR 1.691, *P* = 0.023). In addition, fasting glucose was negatively correlated with the decline rate of nocturnal SBP (r = -0.095, *P* = 0.029). Reverse-dipper pattern of BP in ABPM may be independently associated with type 2 diabetes in patients with hypertension.

Type 2 diabetes is a multifactorial, chronic metabolic disorder, which is characterized by hyperglycemia over a prolonged period^1^. Untreated type 2 diabetes causes cardiovascular, renal and retinal pathological changes and has become crushing burdens impacting the life quality of patients^2^,^3^. It was widely recognized that a large amount of patients with type 2 diabetes have concomitant hypertension, reflecting substantial overlap in etiology and pathological mechanism, such as obesity, insulin resistance and hyperglycemia^4^. It has been shown that type 2 diabetes may influence blood pressure (BP) due to sympathetic excitation by hyperinsulinemia and activated intrarenal renin–angiotensin system by hyperglycemia^5^. On the other hand, BP and its circadian variations also serve as risk factors for type 2 diabetes. A study has reported that the high level of BP in hypertensive patients is positively associated with fasting plasma insulin secretion^6^.

Circadian BP patterns previously were divided into dipper (10% to 20%), extreme dipper (>20%) and nondipper (<10%) based on the nocturnal fall of BP^7^,^8^. A large cross-sectional study on Spanish population showed that nondipper pattern of circadian BP was more common in patients with than without type 2 diabetes and the risk of cardiovascular events was also significantly higher than those patients with dipper pattern^9^. In the growing number of studies, reverse dipper BP pattern, a variant of nondipper with higher average nighttime BP than daytime BP, was shown not only closely associated with severe renal damage and cardiovascular injuries^10^, but also related to inflammation and regarded as an independent predictor of graft outcome in patients with chronic kidney disease^11^. Besides, we have shown BP reverse dipping may contribute to the initiation of carotid plaque formation and lacunar infarction in hypertensive patients^12^,^13^. However, little is known about the relationship between type 2 diabetes and elevated nocturnal SBP. Therefore, we conducted this study to investigate the relationship between reverse dipper BP pattern and type 2 diabetes.

## Results

### Study Population

In our study, a total of 134 patients (25.2%) were type 2 diabetes. The mean age of hypertensive patient with type 2 diabetes was 60.5 ± 12.6 years. There was no significantly statistic difference between type 2 diabetes group and control group in demographic, lipid profile and ambulatory blood pressure monitoring (ABPM) results (Table 1). Moreover, no remarkable difference was observed in terms of the prevalence of diabetes and the circadian BP patterns between males and females.

### Reverse Dipper BP Pattern Was Correlated With Type 2 Diabetes

**Figure 1:**
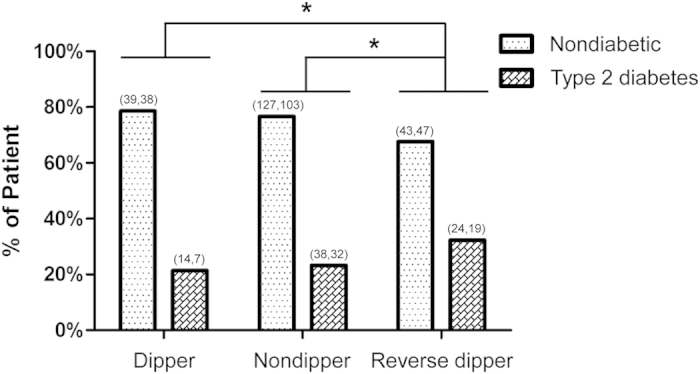
The distribution of diabetic and non-diabetic patients in each circadian BP pattern group. The difference between dipper and reverse-dipper pattern, nondipper and reverse-dipper pattern are statistically significant (*P* = 0.046 and *P* = 0.033, respectively). Patients with reverse-dipper pattern showed the lowest prevalence of non-diabetic and the highest prevalence of type 2 diabetes. (X, Y) above column indicate that (The number of Males, The number of Females). *indicates statistic significance between two group.

**Figure 2:**
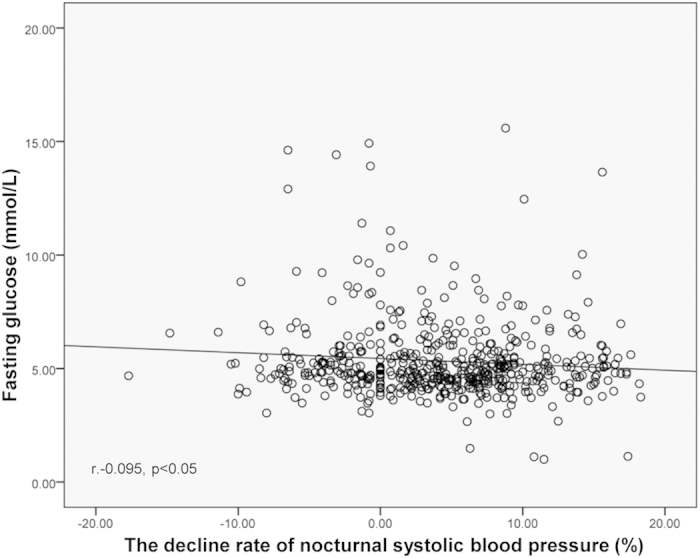
The correlation of fasting glucose with the decline rate of nocturnal SBP.

The distribution of hypertensive patients with or without type 2 diabetes between each circadian BP pattern group was analyzed using chi-squared test. The difference between dipper and reverse dipper (*P* = 0.046), nondipper and reverse dipper (*P* = 0.033) were statistically significant. Patients with reverse-dipper pattern had the highest prevalence of type 2 diabetes (Fig. 1). After univariate and multivariate logistic regression analysis for type 2 diabetes, reverse dipper (OR = 2.067, *P* = 0.024) and nondipper (OR = 1.637, *P* = 0.039) were found to be correlated with type 2 diabetes compared with dipper (Table 2). In addition to this, bivariate correlation analysis revealed that fasting glucose was negatively correlated with the decline rate of nocturnal SBP (r = −0.095, *P* = 0.029) (Fig. 2).

### Type 2 Diabetes Affected Circadian Pattern of BP

Our data indicated that there was no significantly difference in 24 h-SBP, 24 h-DBP, daytime SBP, daytime DBP, nighttime SBP and nighttime DBP between type 2 diabetes and non-diabetic patients (Table 1). Multivariate logistic regression analysis was carried out to investigate further and showed the age of patients (OR = 1.028, *P* = 0.001) and type 2 diabetes (OR = 1.691, *P* = 0.023) were significantly different between reverse dipper and non-reverse dipper BP pattern. Besides, patients with TG levels from 1.7 to 2.3 mmol/l (OR = 3.522, *P* = 0.001) or more than 2.3 mmol/l (OR = 3.524, *P* < 0.001) were associated with the presence of reverse-dipper compared with TG levels less than 1.7 mmol/l (Table 3).

## Discussion

Numerous studies have provided evidences that nondipper and other circadian BP patterns may constitute risk factors for left ventricular hypertrophy, congestive heart failure, myocardial infarction, cerebrovascular disease, microalbuminuria, and vascular dementia^14^,^15^,^16^,^17^. It was reported that the blunted nocturnal decline of SBP during 00:00–04:00 AM was associated with chronically elevated blood glucose in a small population of African men^18^. In addition, a large cross-sectional study on Spanish has shown that the prevalence of non-dipping BP pattern was significantly higher in patients with than without diabetes^9^. Our pilot data also found that circadian decline rate of SBP was closely related to the prevalence of diabetes (OR 0.963, CI 0.934–0.993, *P* = 0.015) (not shown) and fasting glucose was negatively correlated with the decline rate of nocturnal SBP (r = −0.095, *P* = 0.029). Flores *et al*.^19^ also showed that nondipping status was common in normotensive, but severely obese patients and the glucose tolerance abnormalities were the primary variables associated with the impaired nocturnal BP fall^9^.

Interestingly, different from other reports on the important prognostic value of nondipper pattern of BP, our results revealed that reverse-dipper pattern, which has higher average nighttime BP than daytime BP and used to be categorized as non-dipper, might play an independently detrimental role on type 2 diabetes (OR = 2.067, *P* = 0.024). On the other hand, reverse dippers might have a higher risk of type 2 diabetes (32.3%) compared with dippers (21.4%) and nondippers (23.3%). In addition, the patients with type 2 diabetes had a significantly higher prevalence of reversed dipper BP pattern compared with the patient without type 2 diabetes (OR = 1.691, P = 0.023).

It seems there may be a link between these results and our previous findings on BP reverse dipping in hypertensive patients. We have revealed that reverse dipper BP pattern might significantly promote the development of carotid plaque^12^ and lacunar infarction^13^, which are recognized as very common complications of type 2 diabetes. Although the pathophysiological mechanism still remain obscure, BP reverse dipping and type 2 diabetes may lead to or/and affect each other, or even connected with carotid plaque and lacunar infarction by common neuroendocrine risk factors contributing to all of them. It was demonstrated that when insulin sensitizer, such as pioglitazone, was used, nocturnal BP declines could be restored, because insulin resistance might play an important role on the development of abnormal circadian BP rhythms. Additionally, hyperinsulinemia may activate mitogen-activated protein kinase pathway, which leads to smooth muscle cell proliferation, increased stiffness and elevated BP variations^19^.

Here presented is a population-based study which included a relatively large sample size. We focused on the Chinese hypertensive patients with BP reverse dipping due to lack of related research. We also excluded patients with antihypertensive treatment or suffering from secondary hypertension, which may affect circadian pattern of ambulatory BP^12^. To be noticed, our findings should not be extended to different ethnic groups, as the study was launched in Chinese population of hypertension. In addition, multiple ABPM over a longer period across different centers may provide more accurate information regarding visit to visit variations and day to day variations, which will be used in our future clinical trial.

In conclusion, reverse-dipper pattern of ABPM may be independently associated with type 2 diabetes in hypertensive patients. The result of our study also suggested that type 2 diabetes might contribute to reverse-dipper pattern of BP.

## Methods

### Study Population

A total of 531 hypertensive patients including 285 men and 246 women were involved in our study. Information was extracted from our entire in-patient ABPM service database from 2013 and 2014. Hypertension was diagnosed as systolic BP (SBP) ≥140 mmHg and/or diastolic BP (DBP) ≥90 mmHg in casual office recording, or daytime (or awake) SBP ≥ 135 mmHg and/or DBP ≥ 85 mmHg or night-time (or asleep) SBP ≥ 120 mmHg and/or DBP ≥ 70 mmHg in ABPM^20^. Exclusion criteria were made to avoid influences on the BP variations. Consequently, hypertensive patients were excluded if the patients (1) were <18 or >90 years old; (2) were pregnant female; (3) were under antihypertensive treatment; (4) had BP measurements over 160/100 mmHg; (5) had night-work employment; (6) had evidences of acute stroke or myocardial infarction within the past 6 months; (7) had sleep apnea syndrome; (8) had evidence of disease or conditions responsible for secondary hypertension; (9) could not tolerate ABPM; (10) had history of any arrhythmia, congestive heart failure, hepatic failure, kidney failure and significant systemic disease. HbA1c value ≥ 6.5% or previous criteria for fasting glucose (≥126 mg/dL) or 2-hour glucose (≥200 mg/dL) were used for the diagnosis of diabetes mellitus, which was recommended by the 2015 Standards of medical care in diabetes^21^. However, HbA1c was incomplete due to the nature of retrospective cross-sectional study, so fasting glucose was used in the following studies. Blood sample for fasting glucose were harvested between 06:30 and 07:00 AM, after last meal at least 10 h, from an antecubital vein and analyzed using the routine automatic biochemical analyzer.

### ABPM Assessment

Trained investigators observed at least two consecutive clinic BP measurements using a validated automatic oscillometric device (Spacelabs 90207; Spacelabs, Redmond, WA, USA) after the participants had rested in a seating position for ≥10 min. Ambulatory BP was recorded over 24 hours and set to measure every 30 min at daytime (from 7:00 AM to 11:00 PM) and every 60 min at nighttime (from 11:00 PM to 7:00 AM) lasting 24 hours automatically. The monitor was installed on the non-dominant arm between 7:00 AM and 9:00 AM and removed 24 hours later. The patients were asked to take activities as usual and avoid daytime napping and sleep for 6 h to 12 h. The occurrence of unusual events or poor sleep should be noted for further evaluation. We calculated the following values from the 24-h BP profiles: mean 24-h systolic and diastolic values, daytime SBP and DBP, nighttime SBP and DBP. The normal day-night dipping of BP was defined for SBP as 10%–20% reduction in mean BP values at night compared with the daytime values^7^. Values of SBP <70 or >250 mmHg, DBP <40 or >150 mmHg and HR <40 or >150 beats per minute were excluded from the recording. Fewer than 3% of the BP readings were rejected as artifacts on the basis of these criteria^22^. BP patterns of patients in our study were divided into dipper (10% to 20% SBP fall), non-dipper (0% to 10% SBP fall), extreme-dipper (>20% SBP fall) and reverse-dipper (<0% SBP fall), according to the range of the nocturnal SBP dip^7^,^8^,^23^. Due to the lack of patients of extreme-dipper pattern, we only investigated the relationships between diabetes and dipper, nondipper or reverse dipper.

The study protocol was approved by the Ethics Committee of the Second Affiliated Hospital, Xi’an Jiaotong University, in compliance with the Declaration of Helsinki. All the patients were referred due to standard indications that have been shown to use ABPM for appropriate clinical circumstances^22^. ABPM were carried out for diagnosis of hypertension and assessment of cardiovascular risk in adults with the approved guidelines^16^. All the participants signed written informed consent.

### Statistical Analysis

Descriptive statistics are presented as percentages for discrete variables and mean ± SD for continuous normally distributed variables. To compare ordinal and continuous normally distributed variables between subgroups of circadian BP patterns and type 2 diabetes, Chi-square test and analysis of variance (ANOVA) were employed, respectively. Variables were categorized as follows: Total cholesterol (TC) <5.2, 5.2–6.2, >6.2 mmol/l; Triglycerides (TG) <1.7, 1.7–2.3, >2.3 mmol/l; Low-density lipoprotein cholesterol (LDL-C) <3.4, 3.4–4.1, >4.1 mmol/l; High-density lipoprotein cholesterol (HDL-C) ≤1.5, >1.5 mmol/l. Serum TC, TG and LDL-C levels were classified as normal, moderately elevated and elevated. HDL-C levels were classified as none and elevated^24^. Parameter with statistical significance in univariate models and acceptable collinearity were then included in the multivariate analyses. The relationship between two continuous variables was assessed by bivariate correlation analysis (Pearson’s correlation). Multivariate logistic regression was also employed to analyze the relationship between circadian BP patterns (dipper, nondipper and reverse dipper) and clinical variables. A calculated difference of *P* < 0.05 was considered to be statistically significant. All the data was analyzed using SPSS 18.0 (SPSS Inc, Chicago, IL).

## Additional Information

**How to cite this article**: Sun, L. *et al*. Relationship between blood pressure reverse dipping and type 2 diabetes in hypertensive patients. *Sci. Rep.* **6**, 25053; doi: 10.1038/srep25053 (2016).

## Figures and Tables

**Table 1:** Baseline characteristics of hypertensive patients with and without type 2 diabetes.

Variable	Non-diabetic	Type 2 diabetes	*P* value
Patients, n	397	134	
Age, years	62 ± 13.7	60.5 ± 12.6	0.231
Male/female	209/188	76/58	0.425
Current smokers, n (%)	128(32.2)	38(28.4)	0.451
Fasting glucose (mmol/l)	4.8 ± 0.9	7.1 ± 3.2	<0.001
Triglycerides (mmol/l)	1.8 ± 1.3	2.0 ± 1.4	0.169
Total cholesterol (mmol/l)	4.5 ± 0.9	4.7 ± 1.1	0.087
HDL-C	1.2 ± 0.3	1.2 ± 0.3	0.378
LDL-C	2.7 ± 0.8	2.8 ± 1.1	0.086
VLD-C	0.6 ± 0.6	0.7 ± 0.6	0.276
24 h-SBP, ABPM (mmHg)	134.3 ± 14.4	136.3 ± 12.7	0.121
24 h-DBP, ABPM (mmHg)	79.4 ± 11.1	77.9 ± 9.3	0.151
Daytime SBP, (mmHg)	135.8 ± 14.6	137.3 ± 12.7	0.22
Daytime DBP, (mmHg)	80.5 ± 10.5	78.7 ± 9.4	0.07
Nighttime SBP, (mmHg)	129.2 ± 15.9	132.3 ± 18.2	0.067
Nighttime DBP, (mmHg)	75.4 ± 11.1	74.2 ± 9.9	0.172

Explanatory footnote: ABPM, ambulatory blood pressure monitoring; DBP, diastolic blood pressure; SBP: systolic blood pressure; HDL-C: high-density lipoprotein cholesterol; LDL-C: low-density lipoprotein cholesterol; VLD-C: very low-density lipoprotein cholesterol. *P* < 0.05 was considered to be statistically significant.

**Table 2:** Variables included in the multivariate logistic regression analysis to identify predictors of the type 2 diabetes.

Variables	Odds ratio	95% confidence interval	*P* value
Dipper pattern			
Reverse dipper	2.067	1.103–3.876	0.024
Nondipper	1.637	1.025–2.615	0.039
Dipper	1		
Total cholesterol (mmol/l)			
>6.2	1.714	1.012–2.901	0.045
5.2–6.2	1.146	0.618–2.124	0.665
<5.2	1		
Triglycerides (mmol/l)			
>2.3	2.108	0.967–4.593	0.061
1.7–2.3	2.248	0.960–5.263	0.062
<1.7	1		

Univariate models included in the model are age, sex, smoking, circadian BP pattern, 24 h-SBP, 24 h-DBP, TC, Triglycerides, HDL-C and LDL-C. *P* < 0.05 was considered to be statistically significant.

**Table 3:** Multivariate logistic regression analysis for reverse-dipper pattern.

Variable	Odds ratio	95% confidence interval	*P* value
Age	1.028	1.011–1.045	0.001
Diabetes	1.691	1.076–2.659	0.023
Triglycerides (mmol/l)			
>2.3	3.524	1.782–6.970	<0.001
1.7–2.3	3.522	1.629–7.614	0.001
<1.7	1		

Univariate models included in the model are age, sex, smoking, diabetes, 24 h-SBP, 24 h-DBP, TC, Triglycerides, HDL-C and LDL-C. *P* < 0.05 was considered to be statistically significant.

## References

[b1] Vijan S. Type 2 diabetes. Ann Intern Med. 152, ITC31-15; quiz ITC316, doi: 10.7326/0003-4819-152-5-201003020-01003 (2010). 20194231

[b2] Sarwar N. . Diabetes mellitus, fasting blood glucose concentration, and risk of vascular disease: a collaborative meta-analysis of 102 prospective studies. Lancet. 375, 2215–2222, doi: 10.1016/s0140-6736(10)60484-9 (2010). 20609967 PMC2904878

[b3] Schramm T. K. . Diabetes patients requiring glucose-lowering therapy and nondiabetics with a prior myocardial infarction carry the same cardiovascular risk: a population study of 3.3 million people. Circulation. 117, 1945–1954, doi: 10.1161/circulationaha.107.720847 (2008). 18378618

[b4] Long A. N. & Dagogo-Jack S. Comorbidities of diabetes and hypertension: mechanisms and approach to target organ protection. J Clin Hypertens (Greenwich, Conn.). 13, 244–251, doi: 10.1111/j.1751-7176.2011.00434.x (2011). PMC3746062 21466619

[b5] Ferrannini E. & Cushman W. C. Diabetes and hypertension: the bad companions. Lancet. 380, 601–610, doi: 10.1016/S0140-6736(12)60987-8 (2012). 22883509

[b6] Ferrannini E. . High blood pressure and insulin resistance: influence of ethnic background. Eur J Clin Invest. 21, 280–287 (1991). 1909631 10.1111/j.1365-2362.1991.tb01371.x

[b7] Kario K. . Stroke Prognosis and Abnormal Nocturnal Blood Pressure Falls in Older Hypertensives. Hypertension. 38, 852–857, doi: 10.1161/hy1001.092640 (2001). 11641298

[b8] Routledge F. & McFetridge-Durdle J. Nondipping blood pressure patterns among individuals with essential hypertension: a review of the literature. Eur J Cardiovasc Nur. 6, 9–26, doi: 10.1016/j.ejcnurse.2006.05.001 (2007). 16843730

[b9] Ayala D. E. . Circadian pattern of ambulatory blood pressure in hypertensive patients with and without type 2 diabetes. Chronobiol Int. 30, 99–115, doi: 10.3109/07420528.2012.701489 (2013). 23098178

[b10] Wang C. . Reversed dipper blood-pressure pattern is closely related to severe renal and cardiovascular damage in patients with chronic kidney disease. PLos One. 8, e55419, doi: 10.1371/journal.pone.0055419 (2013). 23393577 PMC3564807

[b11] Ibernon M. . Reverse dipper pattern of blood pressure at 3 months is associated with inflammation and outcome after renal transplantation. Nephr, Dial, Transpl. 27, 2089–2095, doi: 10.1093/ndt/gfr587 (2012). 22015441

[b12] Yan B. . Blood Pressure Reverse-Dipping is Associated With Early Formation of Carotid Plaque in Senior Hypertensive Patients. Medicine. 94, e604, doi: 10.1097/md.0000000000000604 (2015). 25761180 PMC4602459

[b13] Yan B. . Reverse-dipper pattern of blood pressure may predict lacunar infarction in patients with essential hypertension. Eur J Neurol. 22, 1022–1025, doi: 10.1111/ene.12659 (2015). 25614275

[b14] Obayashi K. . Nocturnal urinary melatonin excretion is associated with non-dipper pattern in elderly hypertensives. Hypertens Res. 36, 736–740, doi: 10.1038/hr.2013.20 (2013). 23575383

[b15] Sander D., Kukla C., Klingelhofer J., Winbeck K. & Conrad B. Relationship between circadian blood pressure patterns and progression of early carotid atherosclerosis: A 3-year follow-up study. Circulation. 102, 1536–1541 (2000). 11004145 10.1161/01.cir.102.13.1536

[b16] Hermida R. C. Ambulatory blood pressure monitoring in the prediction of cardiovascular events and effects of chronotherapy: rationale and design of the MAPEC study. Chronobiol Int. 24, 749–775, doi: 10.1080/07420520701535837 (2007). 17701685

[b17] Kadoya M. . Plasma brain-derived neurotrophic factor and reverse dipping pattern of nocturnal blood pressure in patients with cardiovascular risk factors. PLos One. 9, e105977, doi: 10.1371/journal.pone.0105977 (2014). 25153796 PMC4143316

[b18] Lammertyn L., Schutte A. E. & Schutte R. Blood glucose and nocturnal blood pressure in African and Caucasian men: The SABPA study. Diabetes Res Clin Pr. 93, 235–242, doi: 10.1016/j.diabres.2011.05.011 (2011). 21632140

[b19] Flores L., Janka M., Canivell S., Jimenez A. & Vidal J. Glucose abnormalities associated with impaired nocturnal fall in blood pressure in normotensive severely obese patients. Diabetes Res Clin Pr. 101, 153–158, doi: 10.1016/j.diabres.2013.05.008 (2013). 23800572

[b20] Mancia G. . 2013 ESH/ESC Guidelines for the management of arterial hypertension: the Task Force for the management of arterial hypertension of the European Society of Hypertension (ESH) and of the European Society of Cardiology (ESC). J Hypertens. 31, 1281–1357, doi: 10.1097/01.hjh.0000431740.32696.cc (2013). 23817082

[b21] American Diabetes Association. Standards of medical care in diabetes–2015. Diabetes Care 38 Suppl 1, S1–S89 (2015).

[b22] Pickering T. G., Shimbo D. & Haas D. Ambulatory blood-pressure monitoring. New Engl J Med. 354, 2368–2374, doi: 10.1056/NEJMra060433 (2006). 16738273

[b23] Grassi G. . Adrenergic, metabolic, and reflex abnormalities in reverse and extreme dipper hypertensives. Hypertension. 52, 925–931, doi: 10.1161/HYPERTENSIONAHA.108.116368 (2008). 18779438

[b24] Report of the National Cholesterol Education Program Expert Panel on Detection, Evaluation, and Treatment of High Blood Cholesterol in Adults. The Expert Panel. Arch Intern Med. 148, 36–69 (1988). 3422148

